# Case reports of one-lung ventilation using Fuji Uniblocker bronchial blockers for infants under one-year-old in uniportal video-assisted thoracoscopic surgery

**DOI:** 10.1097/MD.0000000000026325

**Published:** 2021-07-09

**Authors:** Szu-Ling Chang, Chih-Hung Lai, Guan-Yu Chen, Chia-Man Chou, Sheng-Yang Huang, Yung-Ming Chen, Tsun-Jui Liu, Hui-Chin Lai

**Affiliations:** a Department of Anesthesiology, Taichung Veterans General Hospital, Taichung; b Departments of Medicine and Surgery, National Yang-Ming University School of Medicine, Taipei; c Department of Medicine and Cardiovascular Center, Taichung Veterans General Hospital, Taichung; d Department of Anesthesiology, Kaohsiung Medical University Hospital, Kaohsiung, Taichung; e Division of Pediatric Surgery, Department of Surgery, Taichung Veterans General Hospital; f National Chung-Hsing University, Taichung, Taiwan.

**Keywords:** one-lung ventilation, surgery, thoracoscopic, uniblocker, uniportal

## Abstract

**Introduction:**

Uniportal video-assisted thoracoscopic surgery (VATS) for various pulmonary diseases provides advantages of less postoperative pain and earlier post-operative recovery over traditional open surgery. The inherent limitation of this surgical modality in manipulation of surgical instruments renders intra-operative one-lung ventilation a requisite to increase the substantially restricted working space and thus visibility of the surgical filed.

**Patient concerns:**

Patient 1, an 8-month-old, 9-kg, and 70 cm-in-height male infant was diagnosed as congenital pulmonary airway malformation (CPAM) over left lower lobe.

Patient 2, a 9-month-old, 8-kg and 72 cm-in-height male infant was diagnosed as CPAM over right lower lobe.

Patient 3, an 8-month-old, 8-kg and 67 cm-in-height female infant was diagnosed as CPAM over left lower lobe.

This facilitating one-lung ventilation yet was rarely conducted in infants under one year of age for the extremely small body size, the unavailability of dedicated tools, and therein the very tough techniques demanded.

**Diagnosis:**

Infants with congenital cystic adenomatoid malformation

**Interventions:**

Here we report three infants of less than one year of age in whom one-lung ventilation was successfully achieved by intraluminal use of 5-Fr Fuji Uniblocker Bronchial Blocker devices and in turn assisted the completion of uniportal VATS for congenital cystic adenomatoid malformation in unilateral lungs.

**Outcomes:**

Three infants received VATS under uniblocker smoothly. Patient 1 had two episode of balloon dislodgement and desaturation and solved by re-insertion. And he had subglottic tracheal stenosis which treatment with laser coagulation. Patient 2 had overall blood loss 80 ml. Patient 3 had one episode of desaturation after stapling the bronchus and fiberoptic bronchoscope revealed obstruction by blood and secretion which solved by suction.

**Conclusion:**

In conclusion, OLV in infants undergoing uniportal VATs could be successfully achieved by Fuji 5 Fr Uniblocker bronchial blockers for as long as 4 hours, as exemplified by our three cases, and balloon poor sealing and dislodgment can be immediately solved by bronchoscope-guided re-positioning without compromising surgical proceeding or outcome.

## 1 Introduction

Uniportal video-assisted thoracoscopic surgery (VATS)^[1,2]^ for management of pulmonary diseases offers the advantages of minor iatrogenic musculoskeletal trauma, less postoperative pain and shorter hospital stay over conventional open surgery^[5]^ as well as 3-port VATS.^[3,4]^ These favorable characteristics make this minimally invasive surgical modality rationally beneficial to pediatric patients of insufficient physical dependence and immature body build-up to smooth their hospital course and reduce future incidence of surgical trauma-related chest deformity. Nonetheless, completion of this surgery requires one-lung ventilation and contralateral lung collapse to compensate for the inherently limited surgical views and acute stapling angles,^[2,6]^ yet this procedure remains extremely challenging for infants younger than one year of age due to the tiny body size and the lack of dedicated devices, as currently available smallest (26 Fr) double- lumen endotracheal tubes^[7,8]^ and Univent tubes (7.5–8.0 mm)^[8,9]^ are both excessively too large for these infant patients. Uniblocker bronchial blocker is a unique device that can achieve one-lung ventilation through a readily placed endotracheal tube. The smaller version of this blocker (5Fr), may accommodate the bronchus of infants and induce one-lung collapse in such small patients. Here we reported 3 infants of unilateral congenital cystic adenomatoid malformation undergoing uniportal VATS in whom one-lung ventilation could be established using 5-Fr Fuji Uniblocker Bronchial Blocker tubes, which were proven feasible to finish the otherwise very tough procedures in such little patients. Thus, one-lung ventilation could be safely and smoothly performed in infants less than one year of age using the intraluminal use of 5Fr Uniblocker Bronchial Blocker device, and in turn facilitates successful accomplishment of Uniportal VATS uneventfully. Episodes of balloon dislodgement and hypoxia were also addressed and discussed. All patients had informed consent from their parents.

## 2 Case report

### 2.1 Case 1

**Figure 1:**
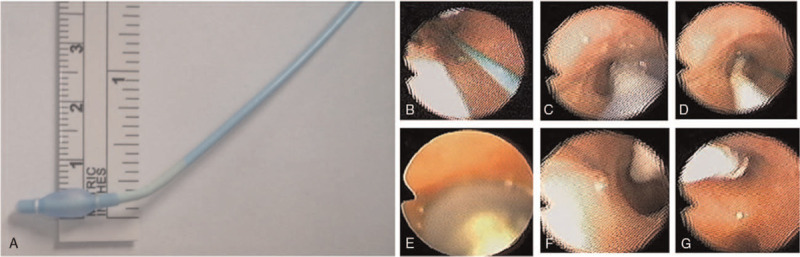
A 5 Fr Uniblocker tube (Fuji Systems Corporation, Tokyo, Japan) was inserted under the guide of a 2.0 mm-out diameter (OD) fiberoptic bronchoscope (Olympus America, Inc., Melville, NY) in the lumen of endotracheal tube. (A) A 5 Fr Uniblocker tube. (B) 2.0 mm fiberoptic bronchoscope in the lumen of endotracheal tube. (C) (D)Inserted Uniblocker under the guide of fiberoptic bronchoscope. (E) Inflate balloon of Uniblocker. (F) Deflate balloon of Uniblocker and advanced a little bit. (G) Uniblocker was checked by the bronchoscope to make sure the upper marker of the balloon was 1–2 mm just beneath the carina.

**Figure 2:**
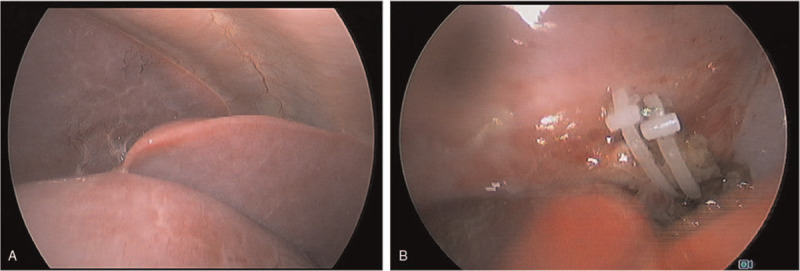
Lung collapse well and perform VATS smoothly. (A) Lung collapse well under the view of thoracoscopy. (B) VATS performed smoothly with clear view.

An 8-month-old, 9-kg, and 70 cm-in-height male infant was diagnosed as congenital pulmonary airway malformation (CPAM) over left lower lobe and scheduled for lobectomy by Uniport VATS as the first case in this institution. After placement of regular monitors, anesthesia was inducted with thiamylal, fentanyl and atracurium, and the patient was then intubated with 5.0 mm uncuffed endotracheal tube. A 5 Fr Uniblocker tube (Fuji Systems Corporation, Tokyo, Japan) was inserted under the guide of a 2.0 mm-out diameter (OD) fiberoptic bronchoscope (Olympus America, Inc., Melville, NY) in the lumen of endotracheal tube. (Fig. 1) The position of Uniblocker was checked by the bronchoscope to make sure the upper marker of the balloon was 1 to 2 mm just beneath the carina. (Fig. 2) The balloon was gradually inflated with air until the breathing sound disappeared over left lung field. Two episodes of balloon dislodgement occurred before the surgical procedure per se, the first when changing position to the left decubitus position, and the second when inserting the surgical instrument into the port, presenting as oxygen desaturation and loss of corresponding lung collapse, respectively. In both episodes the position of Uniblocker was examined by bronchoscope and was adjusted inwards to the proper position. Then the surgical lung remained well collapsed all the time through resection of the left lower lobe, around 4 hours long. The ventilator was set at volume control mode with FiO_2_ 100%, tidal volume 8 ml/kg, I.E. ratio 1:1.5, and respiratory rate 24 /min. End-Tidal CO_2_ was around 28 to 38 mmHg, and SpO_2_ was always above 97% during the rest course of one lung ventilation. At the timepoint when the surgeon stapled the bronchus, the bronchial balloon was deflated and the Uniblocker withdrawn into the tracheal tube to avoid incorporation with the surgical staple line.^[12]^ The whole surgical procedure was then finished smoothly, and two-lung ventilation was resumed, with a total one lung- ventilation period of 245 minutes and blood loss of 20 ml without blood transfusion. The Uniblocker tube was removed immediately after he was sent to the intensive care unit. The entotracheal tube was removed by the patient himself one day later, but was reinserted hours later for O_2_ desaturation. After 10 days of ICU care, the endotracheal tube was successfully weaned. Though subglottic tracheal stenosis occurred at 17 days, deemed to be due to tracheal re-intubation, this problem was solved by laser coagulation surgery, and he was discharged home on day 19 Table 1.

**Table 1:** Demographics and one-lung ventilation parameters of all three infant cases.

	Case 1	Case 2	Case 3
Age (mo)	8	9	8
Gender	male	male	female
Body weight (kg)	9	8	7.9
Lung lesion site	LLL	RLL, RML	LLL
Endotracheal tube	5 Fr, uncuffed	5 Fr, uncuffed	4.5 Fr, cuffed
Two-lung ventilation			
PaO2 (mm Hg)	244.9	352.4	252.4
PaCO2 (mm Hg)	27.6	30.4	30.4
One-lung ventilation			
Duration (min)	245	240	200
PaO2 (mm Hg)		113.2	
PaCO2 (mm Hg)		40.2	
Balloon dislodgement	2 episodes, one during position changing; the other during instrument insertion	1 episode, during instrument insertion	1 episode, during instrument insertion
Hypoxic event	nil	nil	One episode, due to occlusion of right bronchus by blood and secretion

### 2.2 Case 2

A 9-month-old, 8-kg and 72 cm-in-height male infant was diagnosed as CPAM over right lower lobe. He was also scheduled for lobectomy under Uniport VATS. Anesthesia was inducted with thiamylal, fentanyl and cisatracurium, and the patient was intubated with a 5.0 mm uncuffed endotracheal tube. Sevoflurane was used to maintain anesthesia. Then, a 5 Fr Uniblocker tube was inserted through the endotracheal tube into the right main bronchus with the guidance of a fiberoptic bronchoscope, and the balloon of the Uniblocker tube just lay beneath the carina (Fig. 2-B). The balloon volume was titrated gradually until the breathing sound over the right lung field vanished. The three lobes of the right lung collapsed quite well during surgery.

One episode of Uniblocker balloon dislodgement occurred in the beginning of lung manipulation, presenting as inability to ventilate. After confirmed by fiberoptic bronchoscopy, the problem was solved by repositioning of the balloon under fiberoptic bronchoscope guidance, with well lung collapse regained throughout the 4-hr surgical time. The ventilator was set at volume control mode with FiO_2_ 100%, tidal volume 6 ml/kg, I.E. ratio 1:1.5, and respiratory rate 28 /min. End-Tidal CO_2_ was around 33 to 39 mmHg. SpO_2_ was above 95% during the whole 270-minute course of one-lung ventilation. Overall blood loss was 80 ml, and 80 ml of packed red blood cell was transfused. The postoperative course was acceptable. Endotracheal tube was removed on day 3, and he was discharged at 8 days uneventfully.

### 2.3 Case 3

An 8-month-old, 8-kg and 67 cm-in-height female infant was diagnosed as CPAM over left lower lobe. Lobectomy under Uniport VATS was scheduled. After induction of general anesthesia, a 4.5 mm cuffed endotracheal tube was intubated smoothly. Under fiberoscopic guidance, a Uniblocker (5Fr) bronchial blocker was introduced into the left main bronchus. (Fig. 2-C)

When the surgeon inserted the instruments, balloon dislodgment was noted and repositioning was performed using fiberoptic bronchoscope guide. The rest of the procedure was performed smoothly with well left-lung collapse for 200 minutes. The ventilator was set at volume control mode with F_I_O_2_ 100%, tidal volume 6 ml/kg, I.E. ratio 1:1.5, and respiratory rate 25 /min. End-Tidal CO2 was around 24 to 31 mmHg, and SpO2 was above 100% during the whole 200-min course of one-lung ventilation. At the end of stapling the bronchus, the bronchial balloon was deflated for resuming two-lung ventilation. However, failure of ventilation occurred and oxygen desaturation ensued. Fiberoptic bronchoscope inspection revealed obstruction the right bronchus by blood and secretion. After several suctions, ventilation and SpO2 returned to normal. Blood loss was 120 ml, and 100 ml of packed red blood cell was transfused. The rest postoperative course was satisfactory, endotracheal tube was removed 20 hours later, and she was discharged on day 7 without adverse events.

## 3 Discussion

Minimally invasive VATS for treatment of pulmonary diseases has been proven as safe and effective as conventional open lobectomy.^[13]^ Recently, owing to the improvement in surgical instrumentation, uniportal VATS^[1,2]^ has largely replaced the role of 3-port-VATS for less postoperative pain and smaller operative wounds.^[3,4]^ Application of uniportal VATS to infant patients, however, remains challenging to surgeons for the grave limitation in the visibility and the working space of the surgical fields,^[2,6]^ and the lack of dedicated devices to achieve ancillary OLV in such little babies. This study for the first time demonstrated OLV could be safely and effectively achieved with the Fuji 5 Fr intraluminal balloon-based Uniblocker bronchial blocker in three infants to assist completion of uniportal VATS for unilateral lung lesions. Blocker migration had happened unexceptionally the early stage just after balloon inflation yet could be immediately and easily solved without interfering in the surgical procedures or causing complications. These findings thus indicate the feasibility of the 5 Fr Uniblocker bronchial blockers to achieve OLV in infants, and hopefully extend the application of uniport VATS to these otherwise non-indicated little patients.

Manipulation of the airway to adjust pulmonary ventilation and facilitate proceeding of VATS can be conducted in several ways.^[7,8,10,11]^ First, two-lung ventilation with small tidal volumes and artificial pneumothorax is easy to carry out yet^[14]^ poses a risk of cardiovascular compromise due to capnothorax and CO_2_ embolism.^[15,16]^ Second, deep advancement of the single-lumen endotracheal tubes into the bronchus to achieve OLV spares the need of specially designed devices yet may not provide adequate sealing of the bronchus, especially when a smaller, uncuffed endotracheal tube is used.^[7,8,17]^ Third, one-lung ventilation using double-lumen tubes could avoid the shortcoming of the aforementioned strategies. However, double-lumen tubes of the smallest diameter (26#) are only suitable for patients above 8 years of age,^[7,8]^ whereas the smallest pediatric Univent tube has an OD of 7.5 to 8.0 mm and is still too large for infants with a regular tracheal size of 3.5 to 5.0 mm.^[8,9]^ In such circumstances, Bronchial blockers represent the only possible tool to achieve OLV, as they offer various sizes and designs to apply to patients of diverse physical characteristics.^[18]^

Previous studies have used Arndt endobronchial blocker in infants and small children with extraluminal or intraluminal method.^[7,8,10,11]^ The Arndt endobronchial blocker has a wire to loop the fiberoptic bronchoscope then guide the placement of blocker. The Fuji uniblocker as we used to have a pre-bended and flexible shaft that was easily manipulated to the desired bronchus without looping the fiberoptic bronchoscope. The fiberoptic bronchoscope was used to check the position only. This was quite beneficial when there was no small diameter fiberoptic bronchoscope available.

All our three infants had encountered balloon dislodgement during surgical instrument insertion or position changing, as reported elsewhere.^[8,21]^ The dislodgements may be the result of smaller airway and limited margin of safety, and was thought to be related to the learning experience of the anesthesiologist, as two consecutive episodes occurred in the first case whereas only one happened in ensuing cases. These events, however, could be solved easily and immediately by bronchoscope-guided repositioning without interfering in subsequent 3 to 4 hours of surgical course. Similar situations have been reported in nine cases of small children and may need a novel technique to reduce the risk of retrograde dislodgement. This technique, namely placing the tip of the endotracheal tube at the carina and passing the endobronchial blocker (Fogarty or Arndt blocker) through the Murphy eye,^[23]^ however, should not be applied to the Fuji Uniblocker with a pre-shaped tip and torque-control manipulability.

Other potential complications with the bronchial blocker, including airway injuries,^[19,20]^ and bronchial blocker incorporation into staple line during operation^[12]^ did not happen in our cases, though for infants the airways are smaller, shorter^[7,22]^ and thus more prone to damage. The only episode of delayed subglottic tracheal stenosis occurring in our first case was related to repeat endotracheal intubation rather than bronchial blockade so should not be regarded as a complication of the Uniblocker.

Though there was no incidence of hypoventilation or hypercarbia in our three cases, a single episode of hypoxia did happen after deflation of the bronchial balloon and was related to obstruction of the tiny bronchus by blood and secretion. This event of hypoxia or hypoventilation portraits the necessity of intensive monitoring and prompt management for such little patients during OLV, as similar complications have been reported to occur in most of the 15 children under two years of age receiving a tidal volume of 4 to 8 ml/kg,^[24]^ and hypercarbia in 9 of 11 patients under 20 months.^[25]^

## 4 Conclusion

In conclusion, OLV in infants undergoing uniportal VATs could be successfully achieved by Fuji 5 Fr Uniblocker bronchial blockers for as long as 4 hours, as exemplified by our three cases, and balloon poor sealing and dislodgment can be immediately solved by bronchoscope-guided re-positioning without compromising surgical proceeding or outcome.

## Author contributions

**Conceptualization:** Hui-Chin Lai, Guan-Yu Chen, Tsun-Jui Liu.

**Data curation:** Guan-Yu Chen.

**Methodology:** Chia-Man Chou, Sheng-Yang Huang.

**Project administration:** Szu-Ling Chang, Chih-Hung Lai.

**Resources:** Hui-Chin Lai, Chia-Man Chou.

**Supervision:** Hui-Chin Lai, Tsun-Jui Liu.

**Validation:** Hui-Chin Lai, Yung-Ming Chen.

**Writing – original draft:** Szu-Ling Chang.

**Writing – review & editing:** Chih-Hung Lai, Tsun-Jui Liu.
